# Prevalence and Risk Factors of Neuropsychiatric Symptoms in Institutionalized Patients with Parkinson’s Disease in Taiwan: A Nationwide Observational Study

**DOI:** 10.3390/healthcare11020258

**Published:** 2023-01-13

**Authors:** Yang-Pei Chang, Ching-Fang Chien, Sun-Wung Hsieh, Ling-Chun Huang, Chung-Fen Lin, Chih-Cheng Hsu, Yuan-Han Yang

**Affiliations:** 1 Department of Neurology, Kaohsiung Medical University Hospital, Kaohsiung Medical University, Kaohsiung 807, Taiwan; 2 Graduate Institute of Medicine, College of Medicine, Kaohsiung Medical University, Kaohsiung 807, Taiwan; 3 Neuroscience Research Center, Kaohsiung Medical University, Kaohsiung 807, Taiwan; 4 Department of Neurology, Kaohsiung Municipal Ta-Tung Hospital, Kaohsiung Medical University Hospital, Kaohsiung 801, Taiwan; 5 Department of Neurology, Kaohsiung Municipal SiaoGang Hospital, Kaohsiung Medical University Hospital, Kaohsiung 812, Taiwan; 6 Institute of Population Health Sciences, National Health Research Institutes, Miaoli 350, Taiwan; 7 Department of Family Medicine, Min-Sheng General Hospital, Taoyuan 330, Taiwan; 8 Department of Health Services Administration, China Medical University, Taichung 404, Taiwan; 9 National Center for Geriatrics and Welfare Research, National Health Research Institutes, Yunlin 632, Taiwan; 10 Department of and Master’s Program in Neurology, Faculty of Medicine, Kaohsiung Medical University, Kaohsiung 807, Taiwan

**Keywords:** neuropsychiatric symptoms, prevalence, risk factors, Parkinson’s disease, nursing home

## Abstract

Neuropsychiatric symptoms (NPSs) are known to be frequent in Parkinson’s disease (PD) with great impacts on the quality of life, but reports about the prevalence in institutions are few. Our aim was to investigate the prevalence of and risk factors for NPSs in institutionalized patients with PD in Taiwan. The National Health Research Institute executed a cross-sectional, community-based, observational study on residential long-term care service institutions. The diagnosis of PD was determined by physicians with the estimated Hoehn and Yahr stage of PD according to the EQ-5D-5L questionnaire. A total of 370 patients with PD (80.1 ± 9.94 years old, 55.1% females) were included, and 139 (37.6%) had more than one NPS in the prior 3 months. The top three NPSs were nighttime behavior (65 (17.6%)), depression (53 (14.3%)), and fear/anxiety (49 (13.2%)). There were no differences between those with NPS and those without NPS in terms of age, gender, education, Mini-Mental State Examination, or Hoehn and Yahr stage. However, multivariate logistic regression analysis showed that genitourinary disease (odds ratio (OR) = 3.13; 95% confidence interval (95%CI) = 1.77–5.51) and psychiatric disorders (OR = 5.18; 95%CI = 3.09–8.69) may be associated with increased risk of NPSs. Increased physical restraint was observed in residents with advanced PD. Genitourinary disease and psychiatric disorders appear to increase the risk of NPSs in institutionalized residents with PD.

## 1. Introduction

Neuropsychiatric symptoms (NPSs), one of the most common types of non-motor symptoms in Parkinson’s disease (PD), include depression, anxiety, apathy, psychosis hallucinations, and impulse control disorders [1,2,3]. Longitudinal studies in the United Kingdom and Korea have revealed that NPSs might also predict an increased incidence of motor progression [4,5]. Most NPSs are more prevalent in PD with cognitive impairment or dementia during the disease [5,6,7]. Such symptoms are considerably associated with poor quality of life of both patients and caregivers and increased costs of care, which may lead to early placement in nursing homes [1,8]. Furthermore, NPSs may be associated with the increased use of physical restraint in nursing homes, which leads to morbidity and death [9,10]. Their prevalence may increase from mild to advanced PD [6]. Previous studies have shown a lack of data about the prevalence of NPSs in patients with PD in nursing homes [11,12]. Thus, our study aimed to determine the prevalence of and possible risk factors for NPSs in institutionalized individuals with PD. A better understanding of the prevalence and essential profiles of NPSs may be helpful for patients with PD and their caregivers in order to improve the quality of life and reduce the caregiver’s burden. Furthermore, the information may provide the reference to the policy of long-term care.

## 2. Materials and Methods

### 2.1. Patients

The Taiwan National Health Research Institute conducted a cross-sectional, community-based, observational study in residential long-term care service institutions in 22 counties and cities in Taiwan from July 2019 to February 2020 to evaluate institutional residents with cognitive impairment and parkinsonism. A detailed description of the selection procedure has previously been published and reviewed [13]. An estimated 6459 patients in 299 care service institutions, including 164 welfare institutions for the elderly, 125 nursing homes, and 10 veteran’s homes in Taiwan, were reviewed. A total of 370 patients were diagnosed with PD after being assessed by neurologists and psychiatrists. We recorded their demographic information, cognition and disability assessments, NPSs, and past medical history.

### 2.2. Study Protocol

The survey was conducted by the National Health Research Institute in Taiwan. The study included two stages. Well-trained interviewers conducted the first stage from July 2019 to November 2019. Interviewers completed the survey for NPSs, Mini-Mental State Examination (MMSE), activity of daily living (ADL), instrumental activity of daily living (IADL), and past medical history assessments before admission to institutions during the case visit. The second stage was conducted by neurologists or psychiatrists with caring service experience for patients with dementia from December 2019 to February 2020. The specialists also evaluated institutional residents with abnormal MMSE scores in the first stage again. All the interviewers or specialists participated in a training course before the survey to achieve a consensus and reduce inter-individual bias in evaluation. The study adhered to patient privacy regulations. The Institutional Review Board and the Research Ethics Committee of the National Health Research Institute (EC1080502) approved the study.

### 2.3. Diagnosis and Clinical Evaluation

A neurologist or psychiatrist interviewed and examined the patients. A semi-structured interview was administered to both the caregiver and patient to collect information on disease history and previous drug therapy. We estimated the Hoehn and Yahr (H & Y) stages according to the EQ-5D-5L questionnaire to improve the diagnostic consistency. Prior studies have showed a high correlation between the H & Y stages and the summary index of the EQ-5D-3L [14,15]. There was no difference between the summary index between the EQ-5D-3L and EQ-5D-5L [15].

### 2.4. Evaluation of Cognition and Disability

The MMSE was used to evaluate cognitive function. We assessed the disability among institutional residents using the measures of ADL and IADL [16,17]. To reduce underestimation, ADLs and IADLs were evaluated based on interviews, observations, or tests conducted by well-trained interviewers [18].

### 2.5. Neuropsychiatric Assessment

We recorded NPSs for each recruited patient. We recorded each patient’s cognitive status, behaviors, and the types and frequency of NPSs described by at least one caregiver living with the patient. Regarding information provided by a qualified caregiver, the following types of NPSs were recorded: wandering, nighttime behavior, language offensive behavior, physically aggressive behavior, interference behavior, resistance against care, delusions, hallucinations, fear or anxiety, depression, self-harm or suicide, repetitive behavior, attacks on items, and inappropriate or unclean behavior.

### 2.6. Statistical Analysis

We performed statistical analyses with the Statistical Package for the Social Sciences (SPSS, version 18.0, IBM, Armonk, NY, USA). The demographic data of all residents and the presence of NPS types with their frequencies were recorded. We performed one-way ANOVA to compare the differences in NPSs, MMSE scores, IADLs, and ADLs in different H & Y stages. We investigated the risks of NPSs by calculating the odds ratio (OR) and 95% confidence intervals (95% CI) with logistic regression analysis. Model 1 was employed without adjusting for confounding factors. Model 2 was adjusted for age, gender, and education. A *p*-value of less than 0.05 was considered to indicate a statistically significant difference.

## 3. Results

A total of 5751 of the estimated 6549 residents from 266 care service institutions in Taiwan completed the survey. Three hundred and seventy residents were diagnosed with PD. Most residents were in stage 4 (83.2%) and had moderate to severely dependent ADLs and IADLs. Hypertension, diabetes mellitus, and cerebrovascular diseases were the top three diseases in the patients’ past medical histories. Approximately 30% of patients with PD needed physical restraints (Table 1).

Among the 370 patients with PD, 139 (37.5%) patients presented with at least one NPS in the previous three months, and 89 (24.1%) patients developed more than two symptoms (data not shown). The most common symptoms were nighttime behaviors, depression, and fear (or anxiety), and the least common were self-harm (or suicide) and wandering (Table 2).

An advanced PD stage may be associated with worse MMSE scores (Data not shown). There were no significant differences between the two groups considering age, gender, education, H & Y stage, and MMSE score. Indeed, patients with PD with NPSs may have greater cognitive impairment than those without (38.1% versus 25.5%, MMSE score 20) (Table 3).

Univariate logistic regression showed an increased risk of NPSs associated with genitourinary disease, psychiatric disorders, and physical restraint after admission and within one week. Physical restraint within one week failed to increase the risk of NPSs in the multivariate analysis, while genitourinary disease and psychiatric disorders were associated with an increased risk of NPSs (Table 4).

## 4. Discussion

We are the first to describe a distribution of patients with PD and their detailed NPSs in nursing homes in Taiwan. In comparison with our previous study on dementia [13], our findings demonstrate different patterns of NPSs in patients with PD. The top three most frequent NPSs in patients with PD were nighttime behavior (17.6%), depression (14.3%), and fear/anxiety (13.2%). In contrast, in patients with dementia, nighttime behavior (17.9%), resistance against care (13.4%), and depression (12.9%) were the most common. In brief, depression and anxiety are more frequent in patients with PD than in patients with dementia [19]. The dependence of ADLs and IADLs was similar to that in a previous study [18].

Previously, Aarsland et al. first led a study in Norway to explore the prevalence of NPSs in PD with comprehensive clinical and neuropsychiatric assessments including the Neuropsychiatric Inventory (NPI). In that study, the most common symptoms were depression (38%), hallucinations (27%), and anxiety (20%). They found that 36 (26%) participants in nursing homes showed higher NPI scores than those in home dwellings, primarily due to more delusions, hallucinations, and disinhibition. Nevertheless, from their results, there were no clear descriptions of the exact prevalence in nursing homes [11].

Similarly, Kulisevsy et al. conducted a study in Spain to investigate the prevalence of NPSs in PD using the NPI, and experienced movement disorder specialists evaluated patients with PD within 3 months. In that study, the top three clusters of the NPI were anxiety (15.6%), depression (13%), and apathy (12.7%). However, no descriptions of the distribution of patients with PD were given [12]. The prevalence of depression in our study was similar that in a previous cross-sectional study (14.3%) and nested case control study (14.3% vs. 18.3%). The relatively lower incidence may be explained by the overlapping symptoms associated with depression, such as sleep disorders, easy fatigue, or poor attention, which can make it difficult diagnose.

We also found some possible risk factors for NPSs, such as a past medical history of psychiatric problems and genitourinary disease and physical restraint use. As expected, genitourinary disease and psychiatric problems may contribute to NPSs. The association of previous psychiatric problems with NPS was in line with previous studies. Indeed, comorbid sleep disturbances and anxiety disorder were found be associated with the risk of depression in PD [20]. Dissanayaka et al. found that self-history of psychiatric disturbances may significantly increase the risk of anxiety disorders in PD [21]. Leetjens et al. reported non-specific factors in a cross-sectional study, including female gender, history of anxiety and/or depression, and worse cognitive status, which may be significantly associated with depression in PD [22]. We did not further clarify the psychiatric problem to a specific domain of NPSs. Future study could be focused on finding the association between psychiatric problems and specific NPSs, in addition to depression and anxiety. Genitourinary diseases were also common in PD. Previous studies concerning the impact of genitourinary disease on NPS were few. Rana et al. reported that lower urinary tract dysfunction may increase the risk of anxiety and depression in PD [23]. We did not collect the information of specific genitourinary disease in each patient, and further study may be warranted to validate our findings. Worthy of mentioning is that we observed that increased physical restraints after admission may be associated with higher percentage of NPSs. While 42.9% (estimated H & Y 3) and 35.4% (estimated H & Y 4) patients received physical restraint, only 10% (estimated H & Y 1) and 12% (estimated H & Y 2) of patients have similar conditions (data not shown). Indeed, there are limited studies reporting the link in patients with PD. Previous studies have showed that NPSs might also play a part in quality of life and be determinants of caregiver burden, of which depression and anxiety are two of the most common symptoms [24,25,26,27]. Therefore, the possible explanations of physical restraint as a risk factor for NPSs may be that an increase in depression, anxiety, and nighttime behaviors together with an increased incidence of resistance against care and language offensive behavior may further increase caregiver burden and lead to increased usage of physical restraints. We urge caregivers and clinicians to treat NPSs in patients with PD in residential care. Appropriate pharmacological treatments for NPSs, particularly for depression and anxiety, may be warranted because a previous study showed that physical restraints may also increase morbidities and mortalities in residential inhabitants [28]. As patients with PD patients experienced advanced disease with increasing NPSs, palliative care may be discussed to improve their quality of life [29].

The strength of this study is that it was a large, cross-sectional study using a standardized assessment tool to evaluate NPSs and their risk factors. However, there are some limitations. Possible limitations of our study are the use of the ED-5D-3L to estimate the H & Y stages of PD. Due to the wide distributions of residential institutions and the evaluation of numerous patient questionnaires and extensive medical history, it was not feasible to apply an additional rating scale for PD. However, patients in the advanced stage showed consistent decline in the dependence in ADL and IADL scores and MMSE scores, which were evaluated by well-trained physicians rather than caregivers. MMSE scores were obtained from 126 (37%) of the reported cases, and our results may be interpretated with caution on the contribution of dementia on the development of NPSs in PD. A more detailed investigation of this issue may be warranted in a future study explicitly focusing on caregiver burden and specific NPSs such as depression and anxiety. We did not record the medications and calculate levodopa-equivalent daily dose (LEDD), which may contribute to the development of neuropsychiatric symptoms, such as visual hallucinations or psychosis. Nighttime behavior was one of the most common NPSs in our study. We defined nighttime behaviors as follows: excessive daytime sleepiness, difficulty of sleep initiation, fragmented sleep, or dream-enacting behavior. Whether these patients had REM sleep behavior disorder (RBD), typical for alpha-synucleinopathy, is not known due to the lack of gold-standard polysomnography data. Lastly, we did not define the purpose of physical restraints in our study and our findings should be read with caution: it warrants further study to know the implications.

## 5. Conclusions

In conclusion, for the first time, we showed the prevalence of NPS in institutionalized Taiwanese PD patients. High rates of NPSs and physical restraints emphasized the need for recognition and treating NPSs in PD patients which can lead to the improvement of their quality of life. Our study also suggests that previous history of psychiatric, genitourinary disease may be associated with a higher risk of NPS. Factors associated with this positive association are unclear and warrant future research.

## Figures and Tables

**Table 1:** Demographic features of the PD patients in nursing homes.

Title 1	*n* = 370 (%)
**Age** (Mean ± SD)	80.1 ± 9.94
**Gender** (**female, n** (**%**))	204 (55.1)
Estimated H & Y stage ^a^	
1	30 (8.1)
2	25 (6.8)
3	7 (1.9)
≥4	308 (83.2)
**Education** (**n, %**)	
0	138 (38.3)
1–6	123 (34.1)
≥7	99 (27.5)
**ADL** (Mean ± SD)	19.2 ± 28.2
**ADL dependence**	
Total independence (ADL100)	7 (1.9)
Mild dependence (ADL 90–99)	9 (2.4)
Moderate dependence (ADL60–89)	33 (8.9)
Severe dependence (ADL20–59)	76 (20.5)
Total dependence (ADL0–19)	245 (66.2)
**IADL** (Mean ± SD)	0.77 ± 1.40
**IADL dependence**	
Normal (IADL 8)	1 (0.3)
Mild dependence (IADL6–7)	6 (1.6)
Moderate dependence (IADL3–5)	34 (9.2)
Severe dependence (IADL 0–2)	329 (88.9)
**Physical restraint use**	
Physical restraint within one week	115 (31.1)
Physical restraint after admission	118 (31.9)
**Past medical history**	
Hypertension	207 (56.0)
Diabetes	93 (25.1)
Orthopedic disease	31 (8.4)
Eye disease	17 (4.6)
Cerebrovascular disease	89 (24.1)
Coronary artery disease	55 (14.9)
Atrial fibrillation	9 (2.4)
Cancers	6 (1.6)
Pulmonary disease	58 (15.7)
Digestive disease	63 (17.0)

H & Y stage: Hoehn and Yahr stage; ^a^ Estimated H & Y stage 1: EQ-5D ≤ 0.2, H & Y stage 2: 0.2 < EQ-5D ≤ 0.3, H & Y stage 3: 0.3 < EQ–5D ≤ 0.6, and H & Y stage ≥ 4: 0.6 < EQ-5D ≤ 1.

**Table 2:** Prevalence and frequency of each NPS type in all PD patients.

	Prevalence of NPS Type in Past 3 Months, N (%)		Frequency of NPS in Past 1 Week, N (%)		
	No	Yes	Never	1–3 Days/Week	4–7 Days/Week
Wandering Nighttime behavior Language offensive behavior	357 (96.5)	13 (3.5)	362 (97.8)	8 (2.2)	0
	305 (82.4)	65 (17.6)	313 (84.6)	52 (14.1)	5 (1.4)
	330 (89.2)	40 (10.8)	332 (89.7)	33 (8.9)	5 (1.4)
Physical aggressive behavior Interference behavior	351 (94.9)	19 (5.1)	351 (94.9)	16 (4.3)	3 (0.8)
	351 (94.9)	19 (5.1)	353 (95.4)	12 (3.2)	5 (1.4)
Resistance against care Delusion Hallucination Fear or anxiety	324 (87.6)	46 (12.4)	327 (88.4)	37 (10.0)	6 (1.6)
	329 (88.9)	41 (11.1)	334 (90.3)	27 (7.3)	9 (2.4)
	336 (90.8)	34 (9.2)	341 (92.2)	25 (6.8)	4 (1.1)
	321 (86.8)	49 (13.2)	325 (87.8)	39 (10.5)	6 (1.6)
Depression Self-harm or suicide	317 (85.7)	53 (14.3)	324 (87.6)	43 (11.6)	3 (0.8)
	366 (98.9)	4 (1.1)	367 (99.2)	2 (0.5)	1 (0.3)
Repetitive behavior	334 (90.3)	36 (9.7)	334 (90.3)	22 (6.0)	14 (3.8)
Attacks on items	365 (98.7)	5 (1.4)	365 (98.7)	4 (1.1)	1 (0.3)
Inappropriate or unclean behavior	358 (96.8)	12 (3.2)	360 (97.3)	7 (1.9)	3 (0.8)

**Table 3:** Association of PD severities and MMSE, NPS, IADL, and ADL.

	NPS		
	No *n* = 231 (%)	Yes *n* = 139 (%)	*p*-Value ^a^
**Age** ≤65			0.0629
	14 (6.1)	16 (11.5)	
>65 **Age**	217 (93.9)	123 (88.5)	
			0.2658
≤75 >75 **Gender** Male	56 (24.2)	41 (29.5)	
	175 (75.8)	98 (70.5)	
			0.3168
	99 (42.9)	67 (48.2)	
Female **Education**	132 (57.1)	72 (51.8)	
			0.1306
0	86 (38.9)	52 (37.4)	
1–6	82 (37.1)	41 (29.5)	
≥7	53 (24.0)	46 (33.1)	
**MMSE**			0.4795
Normal (MMSE 27–30)	10 (14.5)	4 (7.0)	
Mild (MMSE 20–26)	14 (20.3)	15 (26.3)	
Moderate (MMSE 10–19)	39 (56.5)	31 (54.4)	
Severe (MMSE 0–9)	6 (8.7)	7 (12.3)	
**Estimated H&Y stage**			0.6801
1	18 (7.8)	12 (8.6)	
2	13 (5.6)	12 (8.6)	
3	4 (1.7)	3 (2.2)	
≥4	196 (84.9)	112 (80.6)	
**ADL dependence**			0.0006 *
Total independence (ADL100)	6 (2.6)	1 (0.7)	
Mild dependence (ADL 90–99)	0	9 (6.5)	
Moderate dependence (ADL 60–89)	19 (8.2)	14 (10.1)	
Severe dependence (ADL 20–59)	43 (18.6)	33 (23.7)	
Total dependence (ADL 0–19)	163 (70.6)	82 (59.0)	
**ADL**			0.1991
Independence	6 (2.6)	1 (0.7)	
Dependence	225 (97.4)	138 (99.3)	
**IADL dependence**			0.5913
Normal (IADL 8)	1 (0.4)	0	
Mild dependence (IADL 6–7)	5 (2.2)	1 (0.7)	
Moderate dependence (IADL 3–5)	20 (8.7)	14 (10.1)	
Severe dependence (IADL 0–2)	205 (88.7)	124 (89.2)	
**IADL**			0.4373
Independence	1 (0.4)	0	
Dependence	230 (99.6)	139 (100)	
**Past medical history**			
Hypertension	126 (54.6)	81 (58.3)	0.4842
Diabetes	57 (24.7)	36 (25.9)	0.7927
Orthopedic disease	23 (10.0)	8 (5.8)	0.1578
Eye disease	9 (3.9)	8 (5.8)	0.4081
Cerebrovascular disease	58 (25.1)	31 (22.3)	0.5408
Coronary artery disease	35 (15.2)	20 (14.4)	0.8416
Atrial fibrillation	5 (2.2)	4 (2.9)	0.6663
Cancers	3 (1.3)	3 (2.2)	0.5261
Pulmonary disease	37 (16.0)	21 (15.1)	0.8158
Digestive disease	40 (17.3)	23 (16.6)	0.8488
Genitourinary disease	29 (12.6)	35 (25.2)	0.0019 *
Psychiatric disorder	22 (9.5)	42 (30.2)	<0.0001*
Intellectual disability	3 (1.3)	3 (2.2)	0.5261
Cerebral palsy	0	1 (0.7)	0.1967
Spinal cord injury	5 (2.2)	1 (0.7)	0.2865
Intractable epilepsy	7 (3.0)	4 (2.9)	0.9333

a: Chi-squared test; *: *p* < 0.005.

**Table 4:** Risks of cognition, PD severity, and past medical history for NPS.

	NPS OR (95% CI)	
**MMSE** Normal (MMSE 27–30) Mild (MMSE 20–26)	Model 1	Model 2
	Ref	Ref
	2.68 (0.68–10.5)	3.16 (0.75–13.2)
Moderate (MMSE 10–19) Severe (MMSE 0–9)	1.99 (0.57–6.95)	2.74 (0.69–10.9)
	2.92 (0.59–14.3)	4.00 (0.71–22.6)
**Estimated H & Y stage** 1 2 3 ≥4		
	Ref	Ref
	1.39 (0.47–4.05)	1.53 (0.51–4.56)
	1.13 (0.21–5.95)	1.28 (0.24–6.89)
	0.86 (0.40–1.85)	1.03 (0.47–2.27)
**ADL dependence**		
Total independence (ADL100)	Ref	Ref
Mild dependence (ADL 90–99)	-	-
Moderate dependence (ADL 60–89)	4.42 (0.48–41.0)	4.86 (0.52–45.4)
Severe dependence (ADL 20–59)	4.61 (0.53–40.1)	5.64 (0.64–49.7)
Total dependence (ADL 0–19)	3.02 (0.36–25.5)	3.79 (0.44–32.3)
**ADL**: dependence vs. independence	3.68 (0.44–30.9)	4.59 (0.54–38.9)
**IADL dependence**		
Normal (IADL 8)	-	-
Mild dependence (IADL 6–7)	-	-
Moderate dependence (IADL3–5)	-	-
Severe dependence (IADL 0–2)	-	-
**IADL**: dependence vs. independence	1.15 (0.68–1.94)	1.26 (0.74–2.13)
**Past medical history**		
Hypertension	1.14 (0.76–1.72)	1.11 (0.73–1.70)
Diabetes	1.16 (0.73–1.84)	1.17 (0.74–1.87)
Orthopedic disease	0.69 (0.32–1.51)	0.70 (0.32–1.56)
Eye disease	1.37 (0.54–3.47)	1.24 (0.49–3.18)
Cerebrovascular disease	0.89 (0.55–1.45)	0.89 (0.54–1.45)
Coronary artery disease	0.96 (0.54–1.70)	0.98 (0.55–1.75)
Atrial fibrillation	1.59 (0.46–5.49)	1.48 (0.43–5.16)
Cancers	1.36 (0.29–6.33)	1.29 (0.27–6.06)
Pulmonary disease	0.87 (0.49–1.53)	0.92 (0.51–1.67)
Digestive disease	1.03 (0.60–1.77)	1.03 (0.58–1.72)
Genitourinary disease	2.43 (1.47–4.03) ^∗^	3.13 (1.77–5.51) ^∗^
Psychiatric disorder	4.95 (2.98–8.22) ^∗^	5.18 (3.09–8.69) ^∗^
Intellectual disability	1.68 (0.38–7.54)	1.36 (0.29–6.30)
Cerebral palsy	3.93 (0.12–125.0)	2.82 (0.09–91.4)
Spinal cord injury	0.38 (0.05–2.93)	0.37 (0.05–2.92)
Intractable epilepsy	0.88 (0.26–2.99)	0.67 (0.19–2.41)

H & Y stage: Hoehn and Yahr stage; MMSE: Mini-Mental State Examination; Model 1: univariate; Model 2: adjusted for age (≤65, >65), gender, and education (0, 1–6, and ≥7), * *p* < 0.001

## Data Availability

The data of the current study are available from the corresponding author on reasonable request.
